# Trends of medical expenditures and quality of life in US adults with diabetes: the medical expenditure panel survey, 2002–2011

**DOI:** 10.1186/s12955-017-0651-7

**Published:** 2017-04-13

**Authors:** Jennifer A. Campbell, Kinfe G. Bishu, Rebekah J. Walker, Leonard E. Egede

**Affiliations:** 1 Center for Patient Care and Outcomes Research, Medical College of Wisconsin, 8701 Watertown Plank Road, Room H3165, Milwaukee, WI 53226 USA; 2 Department of Medicine, Division of General Internal Medicine, Medical College of Wisconsin, 9200 W. Wisconsin Ave, Milwaukee, WI 53226 USA; 3 Center for Health Disparities Research, Department of Medicine, Medical University of South Carolina, 135 Rutledge Avenue, Room 280, MSC 250593, Charleston, SC 29425 USA; 4 Department of Medicine, Division of General Internal Medicine and Geriatrics, Medical University of South Carolina, 171 Ashley Avenue, Charleston, SC 29425 USA

**Keywords:** Medical expenditures, Quality of life, Out-of-pocket cost, Diabetes

## Abstract

**Background:**

Studies indicate a relationship between cost and quality of life (QOL) in diabetes care, however, the interaction is complex and the relationship is not well understood. The aim of this study was to 1) examine the relationship of quartiles of QOL on cost amongst U.S. adults with diabetes, 2) investigate how the relationship may change over time, and 3) examine the incremental effect of QOL on cost while controlling for other relevant covariates.

**Methods:**

Data from 2002–2011 Medical Expenditure Panel Survey (MEPS) was used to examine the association between QOL and medical expenditures among adults with diabetes (aged ≥18 years) *N* = 20,442. Unadjusted means were computed to compare total healthcare expenditure and the out-of-pocket expenses by QOL quartile categories. QOL measures were Physical Component Summary (PCS) and Mental Component Summary (MCS) derived from the Short-Form 12. A two-part model was then used to estimate adjusted incremental total healthcare expenditure and out-of-pocket expenses adjusting for relevant covariates.

**Results:**

Differences between the highest and lowest quartiles totaled $11,801 for total expenditures and $989 for out-of-pocket expenses. Over time, total expenditures remained stable, while out-of-pocket expenses decreased, particularly for the lowest quartile of physical component of QOL. Similar trends were seen in the mental component, however, differences between quartiles were smaller (average $5,727 in total expenses; $287 in out-of-pocket). After adjusting for covariates, those in the highest quartile of physical component of QOL spent $7,500 less, and those in the highest quartile of mental component spent $3,000 less than those in the lowest quartiles.

**Conclusions:**

A clear gradient between QOL and cost with increasing physical and mental QOL associated with lower expenditures and out-of-pocket expenses was found. Over a 10-year time period those with the highest physical QOL had significantly less medical expenditures compared to those with the lowest physical QOL. This study demonstrates the significant individual and societal impact poor QOL has on patients with diabetes. Understanding how differences in a subjective measure of health, such as QOL, has on healthcare expenditures helps reveal the burden of disease not reflected by using only behavioral and physiological measures.

## Background

Type 2 diabetes is the seventh leading cause of death in the U.S. and affects more than 29 million people or 9.3% of the total United States (U.S.) population [1]. It is associated with a number of comorbid conditions including cardiovascular disease, kidney disease and stroke [1]. Type 2 diabetes is the primary cause of kidney failure in approximately 44% of new diagnosed cases, and those with type 2 diabetes are 1.5 times more likely to be hospitalized for stroke compared to those without type 2 diabetes [2]. Type 2 diabetes and the associated complications disproportionately affect ethnic minorities, with non-Hispanic Blacks experiencing higher rates of morbidity and mortality compared to other ethnic groups [1, 3]. Medical expenditures among those diagnosed with type 2 diabetes are estimated to be 2.3 times higher than those without diabetes; and in 2012 the overall direct and indirect cost of diabetes at the national level was an estimated $245 billion [1]. These expenditures are expected to increase as incidence in type 2 diabetes are estimated to more than double by 2050 [4].

Medical expenditures are found to be highest for those with a longer history of diabetes and for those who suffer greater complications compared to those who are newly diagnosed or who have pre-diabetes [5, 6]. In particular, long term complications that require hospital stays and medication expenditures are responsible for high cost in diabetes care [5, 7]. Recent examination of trends in medical expenditures among patients with diabetes also found that expenditures more than doubled over a ten-year period for patients with diabetes compared to expenditures for those without [7]. Other examination of medical expenditures amongst patients with diabetes have found that the treatment of co-morbid conditions adds to the burden of cost, with cardiovascular complications and renal failure increasing the existing cost of diabetes care by an additional 50% [8].

Medical expenditures have been shown to be associated with quality of life (QOL) in patients with type 2 diabetes. While the direction of this relationship is uncertain, as duration of diabetes and costs increase, QOL has been shown to decrease [9–11]. Cost driving factors among patients with diabetes that may influence QOL include obesity and disability [12–19]. For example, using a health utility score, Lee and colleagues found that in patients with diabetes, as BMI increased, health utility score decreased by -0.0079 [16]. In addition, out-of-pocket costs, specifically, have been associated with differing levels QOL in patients with diabetes [20–22]. Piette and colleagues found in a national survey assessing out-of-pocket medication costs for patients with diabetes, that nearly 30% of participants had difficulty paying for food and diabetes specific medications [21]. In addition, 20% reported lack of adherence to medication regimens due to cost of medications. A follow-up investigation found that patients with high out-of-pocket costs experienced worse diabetes outcomes and lower physical and mental QOL compared to patients who did not experience high out-of-pocket costs [20].

As seen in the literature, there is some relationship between cost and QOL in diabetes care, however, the interaction is complex and the relationship is not well understood. For example, a qualitative assessment examining patient perception of QOL and diabetes related treatment and complications found that perception of QOL, in the presence of comorbidities and complex treatment plans, varied by demographic characteristics [23]. This suggests that a greater understanding of the key factors that drive the relationship between cost and QOL are warranted. Moreover, understanding whether QOL and cost change over time is needed. To address this gap in the literature, we used multi-year data from the Medical Expenditure Panel Survey, to 1) examine the relationship of quartiles of QOL on cost amongst U.S. adults with diabetes, 2) investigate how the relationship may change over time, and 3) examine the incremental effect of QOL on cost while controlling for other relevant covariates.

## Methods

### Data source and study population

We used data from 2002–2011 Medical Expenditure Panel Survey Household Component (MEPS-HC) to examine the association between QOL and medical expenditures among adults with diabetes (aged ≥18 years). We identified 20,442 (weighted sample of 18,157,187) US adults with self-reported diabetes from MEPS-HC. The MEPS-HC includes several waves of national surveys of families and individuals, their medical providers, and employers in the U.S. The MEPS sample is drawn from reporting units in the previous year’s National Health Interview Survey (NHIS), a nationally representative sample with oversampling for Black and Hispanics of the U.S. civilian non-institutionalized population [24–26].

To ensure sufficient sample size and robust estimation for our analysis, we pooled 10 years of MEPS data. Because they have a common variance structure, we can ensure compatibility and comparatively of our variables within the complex sample design [7]. Our study accounts for the sampling weights, clustering and stratification design to estimate the nationally representative unadjusted mean and adjusted incremental healthcare expenditure for the U.S. population [26].

The MEPS household survey collects detailed information on individual socio-demographic characteristics, health conditions, healthcare use and expenditures, sources of payment and health insurance coverage [26]. Data collection is designed in such a way that interview frequency validates the responses. The 2002–2011 total direct medical and out-of-pocket expenditures were adjusted to a common 2014 dollar using the consumers price index obtained from the Bureau of Labor Statistics (BLS) [27].

### Measures

#### Variables of interest

The dependent variable in this study is the total direct healthcare expenditures and out-of-pocket expenses for the calendar year of each observation. Total direct healthcare expenditures in MEPS-HC is defined as the sum of direct payments for care provided during the year, including out-of-pocket payments and payments by private insurance, Medicaid, Medicare and other sources [26]. This is composed of office-based medical provider expenditure, hospital outpatient expenditure, emergency room expenditure, inpatient hospital (including zero night stays) expenditure, prescription medicine expenditure, dental expenditure, home health care expenditure and other medical expenses [26]. The out-of-pocket expenses were defined as the amount paid by family for medical provider visit, non-physician services, hospital inpatient stays, emergency room visit, dental visit, home healthcare, and prescription medications. These expenditures included direct payments, deductibles, co-insurance, co-payments, and premiums for services received during the year [28].

The primary independent variables were QOL components, Physical Component Summary (PCS) and Mental Component Summary (MCS) derived from the Short-Form 12 Version 2 (SF-12V2), collected in the same year as expenditures. PCS and MCS scores were calculated according to the standard algorithm and incorporating imputations for some cases with missing data. The scoring algorithms of PCS42 and MCS42 create continuous variables that incorporate information from all 12 questions, with higher scores indicating better QOL [26]. Finally, we converted the continuous score of MCS and PCS into quartiles of equal distribution. We maintained the quartiles defined by the entire US population for both PCS and MCS, despite the analysis using only data from the population of those with diabetes to allow comparison to similar applications in clinical research [29, 30].

### Controlled covariates

All controlled covariates used for analysis were based on self-report. Binary indicators of co-morbidities were based on a positive response to a question “Have you ever been diagnosed with xxx”. Cardiovascular disease (CVD) indicates a positive response to a coronary heart disease, angina, myocardial infarction or other heart diseases. Race/ethnic groups were categorized into: Non-Hispanic White (NHW), Non-Hispanic Black (NHB), Hispanic or others. Education was categorized into: less than high school (≤ grade 11), high school (grade 12) and college or more (grade ≥ 13). Marital status was categorized into: married, non-married and never married. Age was categorized into: 18–44, 45–64 and ≥ 65 years. Census region was categorized into: Northeast, Midwest, South and West. Metropolitan Statistical Area (MSA) is a region with high population density and was used as an indicator of rural/urban residence, with MSA region indicating urban residence. Health insurance was categorized into: private, public only and uninsured at all time in the year. The income level was defined as a percentage of the poverty level and grouped in to four categories: poor (<125%), low income (125% to less than 200%), middle income (200% to less than 400%) and high income (≥400%). Calendar year was grouped in to five consecutive years of 2002/03, 2004/05, 2006/07, 2008/09, 2010/11 for the pooled data.

### Analyses

Descriptive statistics were used to describe QOL quartiles and characteristics of adults with diabetes. Proportions in each quartile were compared over time using chi2 tests for PCS and MCS. Unadjusted means were computed to compare total healthcare expenditure and the out-of-pocket expenses by quartile categories. A two-part model was then used to estimate adjusted incremental total healthcare expenditure and out-of-pocket expenses [31, 32]. In the two-part model, a probit model is estimated for the probability of observing a zero versus positive medical expenditure. Conditional on having positive medical expenditure, a generalized linear model (GLM) was then estimated in the second part [33]. These models have been widely employed in situations where, due to large number of non-users of health services, there are excess zeros in the resource use or cost data and the assumption of normality of the error term is not satisfied [34]. Cost estimates generally have an excess number of zeros for total and out-of-pocket payments, so a two-part model is recommended [33–35]. We confirmed fit of the two-part model and then used the margins command in STATA to calculate marginal effects and their standard errors from the combined first and second parts of the final model [33]. For the two-part model, the use of GLM in the second part has an advantage over log OLS since it relaxes the normality and homoscedasticity assumptions and avoids bias associated with retransforming to the raw scale [33]. To control for confounding, socio-demographic factors including age, sex, race, marital status, education, health insurance and metropolitan statistical area status, region, income level, comorbidities, and time trend were included in the model. We also tested for interactions between quality of life quartiles and time. Finally, we estimated the US burden for QOL based on the unadjusted and adjusted annual estimates.

The Park test was used as a diagnostic test to examine the model fit. The results of the modified Park test verified the use of a gamma distribution with a log link was the best–fitting GLM to get consistent estimation of coefficients and marginal effects of medical expenditure. Multicollinarity was checked for predictors of the two-part model taking in to account the complex survey design. Variance inflation factor (VIF) for all predictors used in the two-part model were indicating no multicollinearity problems. All analyses were performed at person-level using STATA 14 (StataCorp LP College Station, TX).

We used standard pairwise comparison methods of Sidak, Scheffe, Bonferroni and Tukey to compare the pooled mean total healthcare expenditure between QOL quartile categories for PCS and MCS [36, 37]. We compare mean expenditures between five groups for each of PCS and MCS (quartile 1 vs quartile 2, quartile 1 vs quartile 3, quartile 1 vs quartile 4, quartile 2 vs quartile 3, quartile 2 vs quartile 4, quartile 3 vs quartile 4).

## Results

### Demographic characteristics

Table 1 shows the characteristics of adults with diabetes included in the cohort. Out of 186,982 adults in the 10 year pooled data, 20,442 (10.9%) individuals had diabetes. Among adults with diabetes, approximately 50% were in the lowest quartile of health status of PCS (quartile 1) and 30% were in the lowest health status of MCS. PCS did improve over time (*p* = 0.005), however, MCS did not have a statistically significant change over time (*p* = 0.41).

**Table 1:** Sample demographics among adults with diabetes (*n* = 20,442, *N* = 18,157,187)

Variables	Percentage (%)	Standard Errors
PCS		
Quartile 1 (scores 4.56–41.53)	49.9	0.58
Quartile 2 (scores 41.54–51.93)	27.7	0.44
Quartile 3 (scores 51.94–56.15)	15.8	0.39
Quartile 4 (scores 56.17–73.88)	6.6	0.25
MCS		
Quartile 1 (scores 0.77–43.84)	29.9	0.50
Quartile 2 (scores 43.85–52.39)	23.6	0.40
Quartile 3 (scores 52.4–57.18)	19.6	0.39
Quartile 4 (scores 57.19–77.37)	26.9	0.47
Age category		
Age 18–44	13.5	0.40
Age 45–64	46.8	0.67
Age 65–85	39.7	0.67
Gender		
Female	51.0	0.59
Race/ethnicity		
Non-Hispanic White	64.4	0.85
Non-Hispanic Black	15.3	0.62
Hispanic	13.6	0.67
Others	6.7	0.43
Marital status		
Married	58.7	0.65
Non-married^a^	32.3	0.61
Never married	9.0	0.35
Education category		
< High School	26.0	0.57
High School	34.5	0.59
College or more	39.5	0.64
Insurance		
Private	60.9	0.63
Public	31.4	0.58
Uninsured	7.7	0.26
MSA status		
MSA	79.9	1.0
Census region		
Northeast	18.1	0.66
Midwest	21.0	0.72
South	40.0	0.89
West	20.0	0.74
Income category		
Poor income	19.9	0.48
Low income	16.3	0.40
Middle income	30.7	0.53
High income	33.1	0.64
Chronic conditions		
Hypertension	72.8	0.50
CVD	31.7	0.58
Stroke	10.2	0.34
Emphysema	4.9	0.25
Joint pain	55.7	0.58
Arthritis	48.4	0.59
Asthma	13.7	0.38
Count of comorbidites		
No comorbidity	10.4	0.35
≥ 1comorbidity	89.6	0.35
Year category		
Year 2002/03	15.5	0.53
Year 2004/05	18.0	0.45
Year 2006/07	20.4	0.49
Year 2008/09	22.8	0.52
Year 2010/11	23.3	0.58

*N* weighted sample size, *n* unweighted sample size, *%* weighted percentage

^a^Non-married stands for widowed/divorced and separated

### Unadjusted cost differences for QOL

The results of unadjusted cost differences for QOL quartile categories overtime among adults with diabetes are shown in Table 2. The overall mean unadjusted direct healthcare expenditures of PCS decreased as QOL increased: for quartile 1 (lowest QOL) was $17,043 (95% CI $16,366, $17,721), quartile 2 was $8,233 (95% CI $7,739, $8,727), quartile 3 was $5,779 (95% CI $5,395, $6,192), and quartile 4 was $5,242 (95% CI $4,660, $5,825). Similarly, the overall mean unadjusted direct healthcare expenditures of MCS decreased as QOL increased: for quartile 1 (lowest QOL) was $15,572 (95% CI $14,864, $16,281), quartile 2 was $11,729 (95% CI $10,931, $12,527), quartile 3 was $9,955 (CI $9,176, $10,734), and quartile 4 was $8,945 (95% CI $9,325, $10,365).

**Table 2:** Mean and 95% CI unadjusted health care expenditures by quality of life (QOL) categories among adult with diabetes (reported as dollars in 2014)

	Quartile 1	Quartile 2	Quartile 3	Quartile 4
Total expenditure, PCS				
2002/03	$15,660 ($14,596, $16,724)	$8,337 ($7,135, $9,538)	$5,475 ($4,648, $6,302)	$5,649 ($4,531, $6,767)
2004/05	$17,836 ($16,064, $19,609)	$9,134 ($7,466, $10,803)	$5,503 ($4,617, $6,389)	$4,332 ($3,505, $5,159)
2006/07	$17,977 ($16,475–$19,479)	$7,956 ($7,109, $8,803)	$5,917 ($4,901, $6,934)	$4,604 ($3,693, $5,515)
2008/09	$17,184 ($15,892, $18,475)	$8,429 ($7,535, $9,322)	$6,078 ($5,437, $6,719)	$5,970 ($4,458, $ 7,482)
2010/11	$16,397 ($15,209–$17,585)	$7,577 ($6,772, $8,382)	$5,802 ($4,993, $6,610)	$5,398 ($4,087, $6,709)
Pooled sample	$17,043 ($16,366, $17,721)	$8,233 ($7,739, $8,727)	$5,779 ($5,395, $6,192)	$5,242 ($4,660, $5,825)
Total expenditure, MCS				
2002/03	$15,123 ($13,833, $16,414)	$11,348 ($9,755, $12,941)	$9,414 ($7,978, $10,849)	$8,890 ($7,961, $ 9,820)
2004/05	$16,015 ($14,165, $17,866)	$14,327 ($11,279, $17,376)	$10,384 ($8,720, $12,067)	$9,451 ($8,256, $10,645)
2006/07	$15,482 ($14,109, $16,855)	$11,215 ($9,843, $12,588)	$11,467 ($9,173, $13,762)	$10,722 ($9,306, $12,137)
2008/09	$15,743 ($14,115, $17,370)	$11,957 ($10,644, $13,270)	$8,811 ($7,607, $10,014)	$10,691 ($9,407, $11,975)
2010/11	$15,455 ($14,085, $16,825)	$10,269 ($9,053, $11,484)	$9,826 ($8,244, $11,409)	$9,140 (8,147–10,134)
Pooled sample	$15,572 ($14,864, $16,281)	$11,729 ($10,931, $12,527)	$9,955 ($9,176, $10,734)	$9,845 ($9,325, $10,365)
Out-of-pocket expenditure, PCS				
2002/03	$2,520 ($2,362, $2,677)	$1,631 ($1,504, $1,757)	$1,441 ($1,241, $1,642)	$1,478 ($1,094, $1,861)
2004/05	$2,716 (2,395–3,038)	$1,953 (1,647–2,259)	$1,357 (1,191–1,522)	$996 ($771, $1,221)
2006/07	$2,103 ($1,950, $2,257)	$1,590 ($1,436, $1,744)	$1,255 ($1,100, $1,410)	$1,135 ($965, $1,304)
2008/09	$1,875 ($1,692, $2,058)	$1,347 ($1,212, $1,482)	$1,097 ($857, $1,341)	$1,137 ($777, $1,496)
2010/11	$1,551 ($1,410, $1,691)	$1,278 ($1,149, $1,408)	$1,121 ($991, $1,252)	$1,011 ($808, $1,214)
Pooled sample	$2,112 ($2,015, $2,210)	$1,523 ($1,442, $1,605)	$1,238 ($1,152, $1,324)	$1,123 ($995, $1,250)
Out-of-pocket expenditure, MCS				
2004/05	$2,453 ($2,004, $2,901)	$2,341 ($2,003, $2,680)	$1,974 ($1,622, $2,326)	$1,944 ($1,750, $2,138)
2006/07	$1,900 ($1,722, $2,078)	$1,659 ($1,492, $1,827)	$1,793 ($1,615, $1,971)	$1,682 ($1,509, $1,856)
2008/09	$1,758 ($1,534, $1,983)	$1,556 ($1,313, $1,799)	$1,199 ($1,077, $1,321)	$1,557 ($1,323, $1,791)
2010/11	$1,372 ($1,216, $1,528)	$1,357 ($1,227, $1,487)	$1,378 ($1,168, $1,589)	$1,355 ($1,176, $1,534)
Pooled sample	$1,935 ($1,814, $2,055)	$1,733 ($1,633, $1,833)	$1,599 ($1,500, $1,697)	$1,648 ($1,556, $1,740)

Mean unadjusted out-of-pocket expenditures of PCS also decreased as QOL increased: for quartile 1 (lowest QOL) was $1,551 (95% CI $1,410, $1,691), quartile 2 was $1,278 (95% CI $1,410, $1,691), quartile 3 was $1,121 (95% CI $1,410, $1,691), and quartile 4 was $1,011 (95% CI $808, $1,214). The overall mean unadjusted out-of-pocket expenditures of MCS for quartile 1 (lowest QOL) was $1,935 (95% CI $1,814, $2,055), quartile 2 was $1,733 (95% CI $1,633, $1,833), quartile 3 was $1,599 (95% CI $1,500, $1,697), and quartile 4 was $1,649 (95% CI $1,556, $1,740).

According to Sidak, Scheffe and Bonferroni all mean group total expenditures for both PCS and MCS except quartile 3 vs quartile 4 were found to be statistically significant at 95% CI. According to Tukey, pairwise comparison showed that all mean group total expenditures of PCS and MCS were statistically significant at 95% CI with the exception of quartile 2 vs quartile 4 for MCS. The results of pairwise comparison tests showed consistent across the three methods (Sidak, Scheffe, Bonferroni).

### Adjusted incremental cost differences for QOL

Table 3 shows the results of the adjusted two-part GLM on the incremental of total healthcare expenditures and out-of-pocket expenses associated with QOL quartile categories. After adjusting for socio-demographic factors, comorbidities and time trend covariates, the total mean direct healthcare expenditures of PCS for quartile 2 was -$5,313 (95% CI -$6,063, -$4,562), quartile 3 was -$6,994 (95% CI -$7,868, -$6,120), and quartile 4 was -$7,499 (CI 95% -$8,402, -$6,596) when compared to the lowest quartile. Overall, total expenditures increased between 2002/03 and the subsequent two time periods ($851 *p* = 0.01 for 2004/05, and $696 *p* = 0.02 for 2006/07), but were not significantly different from 2002/03 in later years. Compared to the lowest quartile, the adjusted total mean direct healthcare expenditures of MCS for quartile 2 was -$1,914 (95% CI -$2,635, -$1,194), quartile 3 was -$3,104 (95% CI -$3,824, -$2,383), and quartile 4 was -$3,262 (95% CI -$3,907, -$ 2,618). Overall, after accounting for MCS, total expenditures increased between 2002/03 and the subsequent two time periods ($1,315 *p* = 0.002 for 2004/05, and $1,012 *p* = 0.003 for 2006/07), but were not significantly different from 2002/03 in later years.

**Table 3:** Two-part regression model: Incremental effects of healthcare expenditures by QOL categories among adults with diabetes (reported as dollars in 2014)

Variables	Unadjusted Incremental Cost and 95% CI	Adjusted Incremental Cost and 95% CI
Total expenditure, PCS		
Quartile 1 (ref)		–
Quartile 2	-$8,810*** (-$9,595, -$8,027)	-$5,313*** (-$6,063, -$4,562)
Quartile 3	-$11,264 (-$12,038, -$10,491)	-$6,994*** (-$7,868, -$6,120)
Quartile 4	-$11,800 (-$12,699, -$10,902)	-$7,499*** (-$8,402, -$6,596)
Total expenditure, MCS		
Quartile 1 (ref)		–
Quartile 2	-$3,843*** (-$4,870, -$2,815)	-$1,914*** (-$2,635, -$1,194)
Quartile 3	-$5,617*** (-$6,589, -$4,644)	-$3,104*** (-$3,824, -$2,383)
Quartile 4	-$5,727*** (-$6,576, -$4,877)	-$3,262*** (-$3,907, -$2,618)
Out-of-pocket expenditure, PCS		
Quartile 1 (ref)		–
Quartile 2	-$588*** (-$705, -$472)	-$408*** (-$529, -$287)
Quartile 3	-$873*** (-$1,002, -$744)	-$611*** (-$750, -$471)
Quartile 4	-$989 (-$1,146, -$831)	-$670*** (-$839, -$501)
Out-of-pocket expenditure, MCS		
Quartile 1 (ref)		–
Quartile 2	-$202** (-$346, -$56)	-$96 (-$213, $20)
Quartile 3	-$335*** (-$486, -$185)	-$286*** (-$407, -$165)
Quartile 4	-$286*** (-$432, -$140)	-$283*** (-$402, -$164)

Primary outcome variable in this regression model is total healthcare and OUT-OF-POCKET expenditures controlling for age, sex, race/ethnicity, marital status, education, health insurance, MSA, census region, income and a number of comorbid conditions (hypertension, CVD, stroke, emphysema, joint pain, arthritis and asthma)

***level of significance *p* < 0.001

The adjusted mean out-of-pocket expenses of PCS for the second quartile was -$408 (95% CI -529– -287), the third quartile was -$611 (95% CI -$750, -$471), and the fourth quartile was -$670 (95% CI -$839, -$501) relative to the lowest quartile. Overall, after accounting for PCS, out of pocket expenses decreased between the years 2002/03 and later years (-$274 -$496, and -$629 respectively with *p* < 0.001 for 2006/07, 2008/09, 2010/11). Compared to the lowest quartile, the adjusted mean out-of-pocket expenses of MCS for the second quartile was -$96 (95% CI -213– 20), the third quartile was -$286 (95% CI -$407, -$165), and the fourth quartile was -$283 (95% CI -$402, -$164). Overall, after accounting for MCS, out of pocket expenses decreased between the years 2002/03 and later years (-$272 -$525, and -$667 respectively with *p* < 0.001 for 2006/07, 2008/09, 2010/11).

**Fig. 1:**
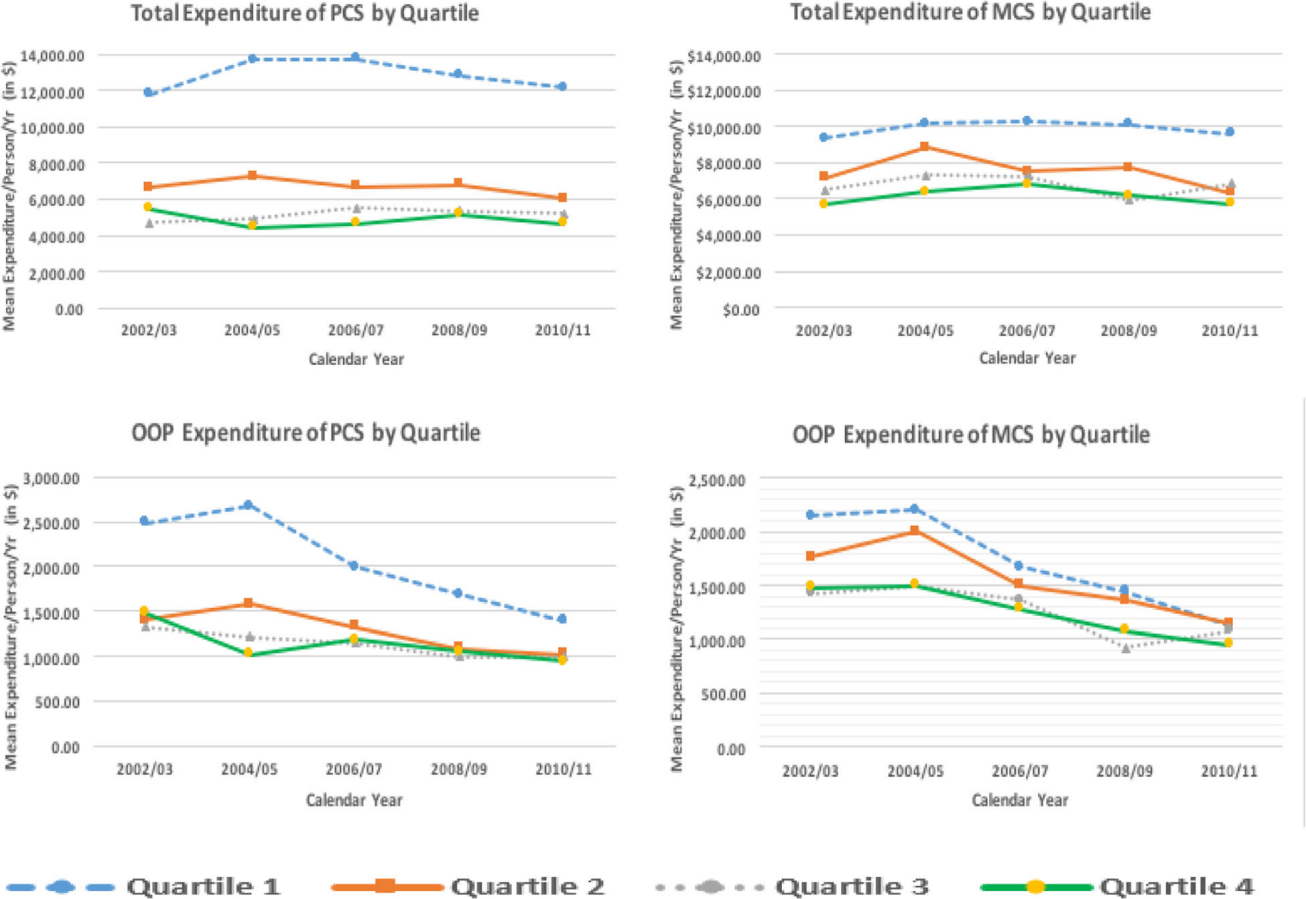
Trends of adjusted healthcare expenditure by QOL categories among adults with diabetes

Figure 1 shows the change overtime for adjusted direct healthcare expenditure and out-of-pocket costs for PCS and MCS presented in Table 3. Total expenditures in PCS remained stable, while out-of-pocket expenses decreased over time, most dramatically for quartile 1. Similar trends were seen in MCS, however, differences between the quartiles were much smaller than in PCS and costs for all quartiles in 2010/11 were nearly the same. Significant interactions did not exist between QOL quartiles and time, indicating the relationship between QOL and expenditure did not change over time.

### Estimated US burden for QOL

Finally, we estimated the annual aggregate cost during 2002–2011 among US adults with diabetes for PCS and MCS in both the lowest and highest quartile. The total annual unadjusted direct expenditure of PCS for the lowest quartile was $124 billion and the highest quartile was $3.7 billion per year. The total annual unadjusted direct expenditure of MCS for the lowest quartile was $68.3 billion and the highest quartile was estimated $34.8 billion per year. After adjustment, the total incremental expenditure of PCS for the lowest quartile was higher at $54.6 billion and the adjusted total incremental expenditure of MCS for the lowest quartile was higher by $14.3 billion in US population, when compared to those with highest quartile.

## Discussion

Overall, this analysis found a clear gradient between QOL and cost with increasing physical and mental QOL associated with lower expenditures and out-of-pocket expenses. Differences between the highest and lowest quartiles totaled $11,801 for total expenditures and $989 for out-of-pocket expenses. Over time, while total expenditures remained stable, out-of-pocket expenses decreased for the lowest quartile of physical component of QOL. Similar trends were seen in the mental component, however, differences between the quartiles were much smaller (on average $5,727 in total expenses and $287 in out-of-pocket). By 2010/11, out-of-pocket expenses for all quartiles of the mental component of QOL were more similar, but differences remained between the lowest and highest quartiles for total expenditures. The relationship between QOL quartiles and expenditures did not change significantly over time; however differences between QOL quartiles and expenditures remained in the 10 year pooled data set. Those in the highest quartile of physical component of QOL spent on average $7,500 less than those in the lowest quartile. Similarly, those in the highest quartile of mental component spent on average $3,000 less than those in the lowest. The incremental effect of out-of-pocket expenses for both components of QOL were comparatively small (less than $1,000). At the societal level, differences in total expenditures between those in the highest and lowest QOL quartiles totaled $54.6 billion for the physical component and $14.3 billion for the mental component (adjusted annual aggregate for the United States).

This study demonstrates the significant individual, clinical, and societal impact poor QOL has on patients with diabetes. Understanding how differences in a subjective measure of health, such as QOL, has on healthcare expenditures helps reveal the burden of disease not reflected by using only behavioral and physiological measures. By investigating both total expenditures and out-of-pocket expenses, this study showed that payments made both by the individual, as well as by private insurance, Medicaid, Medicare and other sources differed based on the patient’s QOL score.

As QOL can be conceptualized to measure aspects of a patient’s health not measurable by biologic measures, such as impact on partners or stress related to treatment, this finding supports the need to consider patient status and preference in the clinical process. The clinical implications of these findings suggest that the healthcare system as a whole, and not the patient alone, is impacted by differences in QOL and should devote resources to addressing it beyond simply treating disease. By integrating treatment methods that address patient QOL within diabetes management, healthcare expenditures may be significantly reduced while also improving clinical outcomes overtime. In addition, consideration of the social determinants of health influencing a patient can influence their health related QOL and in turn their health condition [38, 39]. This study is strengthened by examining trends in cost and QOL overtime. Trends indicate that the relationship between QOL and out-of-pocket expenses is shrinking, which may in part be related to changes in healthcare policy such as the Medicare Modernization Act which enacted prescription drug benefits, Medicare Part D effective in 2006, as well as adoption of the Affordable Care Act in 2010. However, trends were not significant in out of pocket expenditures and the overall difference in total expenditures between those with the highest and lowest QOL remained similar across 10 years.

Other findings examining QOL and medical expenditures have found that the physical component as well as the mental component of QOL predicted cost related medication underuse within diabetes populations [20]. Piette and colleagues demonstrated that across those insured through private insurance, Medicaid, Medicare, and the non-insured, cost related non-adherence in patients with diabetes was nearly 3 times as high among the privately insured with multiple comorbidities and even higher among those insured by Medicaid, Medicare, or no insurance. Furthermore, both physical and mental components of QOL was significantly lower among those with multiple comorbidities [20]. The current findings offer further support that QOL is a key factor that may be influencing patient outcomes and cost within diabetes care and other chronic illness. However, further research is needed to fully understand the direction of this relationship.

This study has some limitations. First, identification of diabetes and comorbid conditions were based on self-report and as such may be subject to participant bias, however previous studies utilizing self-report disease conditions in national data sets have been shown to be reliable measures. Second, estimates of diabetes and other chronic illness may be higher than what is reported in this study due to the exclusion of institutionalized individuals as well as the population living with undiagnosed diabetes. Third, although two-part models are not always necessary when populations have a low number of zero cost, as is often the case for diabetes patients, we examined the proportion of zeros and it was 1% for total expenditures and 3% for out of pocket expenditures. While these numbers are relatively small, the study is designed to extrapolate to the entire US population, which means that small sample numbers translate to large population numbers. Additionally, because there is no information about whether the zero costs are truly non-users of care or those that received free care, we chose to control for possible selection bias using the two-part model. Finally, this data is cross-sectional and therefore cannot speak to causality. While data has been pooled over a ten-year time period to establish trends, these findings cannot be interpreted as longitudinal data.

## Conclusion

In conclusion, this study found a gradient between QOL and cost as seen by the increase in physical and mental components of QOL and lower expenditures and out-of-pocket expenses. The pooled data showed that over a 10-year time period those with the highest physical QOL had significantly less medical expenditures compared to those with the lowest physical QOL. Similar trends were seen for the mental component of QOL with those in the highest quartile paying significantly less than those in the lowest quartile, however difference in expenditures for the physical component of QOL was higher than then the mental component. Findings suggest the healthcare system as a whole is impacted by differences in patient QOL and should take this into consideration through efforts such as integrating treatment and attention to the social determinants of health impacting patients. Clinicians should also be aware that cost is not only related to comorbidities, but also more general well being.
